# Effect of different short-term high ambient temperature on chicken meat quality and ultra–structure

**DOI:** 10.5713/ajas.18.0232

**Published:** 2018-10-26

**Authors:** Minghao Zhang, Lixian Zhu, Yimin Zhang, Yanwei Mao, Mingyue Zhang, Pengcheng Dong, Lebao Niu, Xin Luo, Rongrong Liang

**Affiliations:** 1 Laboratory of Meat Processing and Quality Control, College of Food Science and Engineering, Shandong Agricultural University, Tai’an, Shandong 271018, China

**Keywords:** Poultry Meat, Meat Quality, Cortisol, Protein Solubility, Meat Color, Ultra–structure

## Abstract

**Objective:**

This study investigated the effect of different acute heat stress (HS) levels on chicken meat quality and ultra-structure.

**Methods:**

Chickens were randomly divided into 7 groups to receive different HS treatments: i) 36°C for 1 h, ii) 36°C for 2 h, iii) 38°C for 1 h, iv) 38°C for 2 h, v) 40°C for 1 h, vi) 40°C for 2 h, and vii) un-stressed control group (25°C). Blood cortisol level, breasts initial temperature, color, pH, water holding capacity (WHC), protein solubility and ultra-structure were analyzed.

**Results:**

HS temperatures had significant effects on breast meat temperature, lightness (L*), redness (a*), cooking loss and protein solubility (p<0.05). The HS at 36°C increased L*_24 h_ value (p<0.01) and increased the cooking loss (p<0.05), but decreased a*_24 h_ value (p<0.05). However, as the temperature increased to 38°C and 40°C, all the values of L*_24 h_, cooking loss and protein denaturation level decreased, and the differences disappeared compared to control group (p> 0.05). Only the ultimate pH_24 h_ at 40°C decreased compared to the control group (p<0.01). The pH in 36°C group declined greater than other heat-stressed group in the first hour *postmortem*, which contributed breast muscle protein degeneration combining with high body temperature, and these variations reflected on poor meat quality parameters. The muscle fiber integrity level in group 40°C was much better than those in 36°C with the denatured position mainly focused on the interval of muscle fibers which probably contributes WHC and light reflection.

**Conclusion:**

HS at higher temperature (above 38°C) before slaughter did not always lead to more pale and lower WHC breast meat. Breast meat quality parameters had a regression trend as HS temperature raised from 36°C. The interval of muscle fibers at 24 h *postmortem* and greater pH decline rate with high body temperature in early *postmortem* period could be a reasonable explanation for the variation of meat quality parameters.

## INTRODUCTION

Chickens subjected to high environmental temperatures typically demonstrate a stress response. Heat stress (HS) has long been recognized as one of the prominent environmental elements influencing the poultry industry [1]. After the 20th century, many scholars have pointed out that HS has negative effects on chicken meat color and water holding capacity (WHC) and is associated with occurrence of pale, soft, exudative (PSE)-like meat, which has softer texture, higher lightness and lower WHC [2–4]. Zhu et al [5] found that chickens in summer transportation in China had poor meat color and low WHC. The incidence of PSE-like meat was reported to levels of 20% in summer in China. In North America, PSE-like meat results in about 2 to 4 million US $ lost for each turkey processing plant and in excess of 200 million US $ for the whole turkey industry [6].

Generally, broilers are raised in a constant temperature of 25°C±1°C to keep them in good health. However, they are often exposed to high ambient temperatures during transportation and the lairage period before slaughter in summer. Because most slaughter house lairage areas do not have air conditioning systems, the temperatures can be extremely high during this period. Poultry with ‘stress syndrome’ may exhibit a rapid decline in postmortem muscle pH. Under this condition, light scattering by precipitated sarcoplasmic proteins increases meat paleness, and the shrinkage of the myofilament lattice at a low pH increases reflection at myofibrillar surfaces, which will increase L* value, and decrease WHC values [7]. Previous reports of HS and chicken meat quality have described various effects. Most publications pointed out that HS had negative effects on chicken meat quality [8–11]. McCurdy et al [8] stated that in poultry, PSE-like meat occurrence increased during hot summers. But some experiments also found extreme HS conditions have no influence on meat quality parameters. For example, Northcutt et al [12] found broilers with acute pre-slaughter HS (40°C, 1 h) had no significant changes in chicken meat drip loss or cooking loss (p>0.05). Pan et al [13] found that acute HS (40°C) could increase breast L* value and decrease a* value when broilers were exposed for 1 to 5 h, but these effects disappeared at 10 h. And Tang et al [13,14] also got similar results. Xing et al [15] pointed out that chicken meat quality was better in 4 h transportation groups in summer than those of 0.5 h to 2 h groups before slaughter. Our previous related experiment also found that there were no obvious changes in chicken meat quality when chickens were subjected to 38°C HS before slaughter (data not yet published). However, the number of reports where there were no effects of high temperature HS on chicken meat quality is quite limited.

In this paper, the research was designed to simulate the exposure of broilers to high ambient temperature during transportation and lariage period before slaughter in summer time, and tried to build a system design to investigate the meat quality and ultra-structure changes of chicken breasts in response to different high *antemortem* HS temperatures (36°C, 38°C, and 40°C) and different exposure times (1 h and 2 h). The control group was also in consideration (no HS treatment).

## MATERIALS AND METHODS

### Materials and treatments

All animal experiments were reviewed and approved by the Institutional Animal Care and Use Committee of Shandong Agricultural University (No. 2001002) and performed in accordance with the “Guidelines for Experimental Animals” of the Ministry of Science and Technology (Beijing, China). All surgery was performed according to recommendations proposed by the European Commission [16], and all efforts were made to minimize suffering.

One hundred male broilers (from *Arbor Acres*) were raised in the same temperature conditions (25°C±1°C) for six weeks. Eighty four broilers were then randomly selected and divided into 7 groups (12 broilers in each group), including six HS groups and one control group. Each group consisted of 6 replicates, and every replicate included 2 broilers in one cage. Broilers in the six HS groups received different HS treatments with different temperatures (36°C, 38°C, and 40°C) and time (1 h and 2 h) in an environmentally controlled room as follows: i) 36°C for 1 h, ii) 36°C for 2 h, iii) 38°C for 1 h, iv) 38°C for 2 h, v) 40°C for 1 h, and vi) 40°C for 2 h. A heater-air conditioning system was used to establish and maintain the HS temperature at the required level and the relative humidity was held at 60%±2% in all the treatments. The control group did not receive HS treatment and remained at the normal growing temperature of 25°C±1°C. Broilers were moved to the preparation room one day before the experiment, so that the broilers were adapted to the new environment. Each replicate was moved into the HS treatment room sequentially with 10 min intervals, and all replicates were kept in same cage position as those in the preparation room. Water was offered throughout the HS procedure. After treatment, replicates were moved out for slaughtering according to the move - in sequence. Broilers were slaughtered and bled within 5 min without stunning or shackling according to Wang et al [17]. Then the left and right boneless *pectoralis major* muscles were removed manually by knife - cutting immediately after bleeding. The left breast was stored at 4°C for aging and for temperature and color test. The right breast was used for WHC, pH, protein solubility analysis and ultra-structure analysis.

### Sampling methods

Blood samples (4 mL) were taken from the wing vein before slaughter, then centrifuged at 4°C for 10 min at 2,000×*g*. The supernatant was frozen in liquid nitrogen and stored at −80°C for cortisol concentration assessment. Meat color parameters of left breasts were recorded at 5 min and 24 h *postmortem* and the initial breasts core temperature was also recorded at 5 min *postmortem*. The top (dorsal-superior) part of the right breast was removed by knife at 5 min *postmortem*, trimmed into a regular shape of 2.5 cm×1.5 cm×1 cm (about 50 g), then suspended in paper cups and covered with plastic film and then stored under 4°C for drip loss analysis. The remaining part of the right breast was cut into small cubes immediately, mixed and randomly divided into six parts and put into six small plastic bags respectively and stored at 4°C for pH and protein solubility analysis. At 5 min, 1 h, 2 h, 4 h, and 24 h *postmortem*, one bag was randomly selected for pH sampling at each sampling time, and then frozen in liquid nitrogen and stored at −80°C until use. At 24 h *postmortem*, the last bag was collected for ultra-structure analysis and protein solubility analysis.

### Cortisol concentration assessment

Frozen supernatants of the blood were sent to the Center Hospital of Tai’an city for total cortisol concentration assessment using electrochemical luminescence immunoassay with modular analytics immunoassay analyzer (Elecsys 170, Roche Diagnostics, Basel, Switzerland).

### Meat color measurement

The left breast color was measured at 5 min and 24 h *postmortem* with a colorimeter (SP62, 8 mm diameter measuring aperture, illuminant D65, X-rite, Grand Rapids, MI, USA). Color parameters were described as coordinates L*, a*, and b*, representing lightness, redness, and yellowness, respectively. Color was measured 6 times with different locations of pectoralis muscle and recorded [17,18]. The result was caculated from the average.

### Temperature and pH measurement

The intial core temperature was measured on the top, middle and bottom of the left breasts separately with a digital thermometer (DM6801A, Shenzhen Victor Hi-tech Co. Ltd., Shenzhen, China) at 5 min *postmortem*.

The pH values of the right breast muscles were measured according to Owen et al [2] and Wang et al [17]. One gram of pectoralis muscle was taken from the collected bags at 5 min, 1 h, 2 h, 4 h, and 24 h, as previously mentioned for pH value test. Each 1 g muscle sample was homogenized in 9 mL of 5 mM iodoacetate solution for 60 s by blender (IKA, T18, Staufen, Germany) at medium speed. The pH value was measured by pH meter (SevenGo, Mettler Toledo, Zurich, Switzerland). The pH value and core temperature were both calculated from the average of the three repetitions.

### Drip loss and cooking loss measurement

Each muscle fillet (about 50 g) from the top right breast was weighed at 5 min *postmortem*. After weighing, the fillets were suspended in paper cups along the direction of the muscle fibres, covered with plastic film, and stored at 4°C for 24 h, and then weighed again [17]. The drip loss (%) value was calculated with the following formula:

$$\text{Drip loss }(\%)=[({\text{weight}}_{5\;\text{min}}-{\text{weight}}_{24\;\text{h}})/{\text{weight}}_{5\;\text{min}}]\times 100\%$$

Cooking loss was analyzed according to Wang et al [17] and Chan et al [19]. After weighing again at 24 h *postmortem*, the fillets were cooked individually in plastic bags and immersed in a 75°C water bath until the core temperature reached 70°C. The core temperature was tracked by digital thermometer (DM6801A, Victor Hi-tech Co. Ltd., Shenzhen, China). The cooked samples were chilled and stored at 0°C to 4°C overnight, then were re-weighed and recorded. The cooking loss (%) was calculated as follows:

$$\text{Cooking loss }(\%)=[({\text{weight}}_{24\;\text{h}}-\text{weightafter cooking})/{\text{weight}}_{24\;\text{h}}]\times 100\%$$

### Protein solubility measurement

The solubility of the sarcoplasmic and total proteins was determined according to the method of Joo et al [20]. For sarcoplasmic protein solubility, one ground meat was homogenized with 10 mL 0.025 M K-phosphate, pH 7.2. After shaking at 4°C overnight, samples were centrifuged at 1,500×*g* for 20 min and protein concentration in the supernatants was determined by the bicinchonininc acid method. For total protein solubility, ground meat of 1 g was homogenized with 20 mL 0.1 M phosphate buffer containing 1.1 M KI (pH 7.2). The same procedures for homogenization, shaking, centrifugation, and protein determination were used as described above.

### Transmission electron microscopy

The sample for electron microscopy at 5 min and 24 h post-mortem was collected as described by Li et al [21] according to the procedure described by Luo et al [22]. The cubes of breast muscles in the middle were prefixed in 3% glutaraldehyde solution, washed with 0.1 M phosphate buffer solution, post-fixed with 1% OsO_4_, re-washed by 0.1 M phosphate buffer solution, dehydrated with graded ethanol, and embedded in spur resin. Sections were prepared on Leica ultramicrotome, stained with uranyl acetate and lead citrate, and examined under a JEM-1200EX transmit electron microscope (JEM-1200EX, Tokyo, Japan).

### Statistical analysis

Statistical analysis was performed with Statistical Product and Service Solutions (IBM SPSS Statistics 19) software with general linear model-Univariate. Different HS temperature, HS time and *postmortem* time acted as fixed factors and those of initial core temperature, pH, color, WHC and the solubility of the sarcoplasmic and total proteins were dependent variables. Three-factor analysis of variance was applied in pH values analysis and two-way analysis of variance was applied in the other indexes’ analyses. Post hoc multiple comparisons were used for observed means and Tukey test was used for equal variances separation. Results were reported as means and standard error, and means were considered to differ significantly at p<0.05.

## RESULTS AND DISCUSSION

### Temperature

The HS significantly increased the initial core temperature in the breasts (p<0.01) at 5 min *postmortem* (after being sliced from the body and before chilling; Table 1). But there was no significant difference among three HS treatments (36°C, 38°C, 40°C; p>0.05). The heat exposure time and interaction of HS time with temperature had no significant effect on the initial breast temperature (p>0.05).

### Cortisol concentration

When broilers are subjected to acute HS, the hypothalamo-pituitary-adrenal glandular system is activated. Corticotropin-releasing hormone and arginine vasopressin are released, stimulating the adrenal cortex to release cortisol [23]. The concentration of serum cortisol has been used as a reliable indicator of stress levels [24]. Therefore, the cortisol concentration was tested as an indicator of HS level in this study. Table 1 shows the cortisol level significantly increased from <0.5 n mol/L (below detectable limit) in the control group to >3.73 n mol/L in heat stressed groups (p<0.01). Also, as HS temperature increased, the cortisol level increased significantly (p<0.01). Both 40°C and 38°C groups were significantly higher than 36°C group (Table 1, p<0.01). Time of exposure to HS also increased broilers’ cortisol level from 3.66 nmol/L to 4.25 nmol/L, but there was no significant difference between the two time treatments (1 h or 2 h, p>0.05). The interaction of HS time with temperature had no effect on the cortisol level (p>0.05). Results showed that the *antemortem* high temperature environment significantly affected the blood cortisol level, and the cortisol level increased with HS temperature increased (p<0.01). Soleimani et al [25] also found similar results that broilers under acute HS had a higher cortisol concentration. However, they did not further investigate the effects of different HS temperatures and times on cortisol level.

### pH value

The crossed effect of HS temperature, HS time and *postmortem* time had no significant effect on pH values (p>0.05). However, the HS temperatures, *postmortem* time and the crossed action had significant effect on pH values (Table 2). HS temperatures had a significant effect on pH_1 h_, pH_4 h_, and pH_24 h_ (p<0.01, Table 2) in breast meat, but had no effect on pH_5 min_ or pH_2 h_ (p>0.05, Table 2). The pH_1 h_ in all HS groups was significantly lower than in the control group (pH 6.36; p<0.05). This indicated that HS treatment stimulated the pH decline in the early *postmortem* period which was in agreement with many other reports. For example, Sams [9] pointed out that HS (38°C/32°C, day/night) resulted in faster pH decline rate and lower ultimate pH of turkey *Pectoralis* compared with the control. Sandercock et al [26] also found acute HS (32°C, 2 h) significantly decreased broilers’ breast muscle pH at 15 min *postmortem* and degraded breast quality like drip loss (72 h *postmortem*) and hemorrhage score. It is noteworthy that the pH_1 h_ in the 36°C HS group was significantly lower than those in the 38°C and 40°C groups. No significant difference existed between pH_1 h_ and pH_2 h_ in the 36°C group and there was no difference among all groups at 2 h either. So, it can be concluded that the pH in the 36°C group (6.15) had a greater decline than other groups during the first hour *postmortem*, and it reached to the level of pH_2 h_.

Acute HS also decreased the pH4h and ultimate pH (pH_24 h_). Results of pH_4 h_ and pH_24 h_ showed similar trends. In contrast to the pH_1 h_, the lowest pH_4 h_ and pH_24 h_ was found in the 40°C group, not the 36°C group. The pH_4 h_ in the 40°C group was significantly lower than the control group (Table 2; p<0.01), but there was no difference at 36°C and 38°C, compared with controls (p>0.05). The pH_24 h_ in 40°C group decreased to the lowest values of 5.79 compared with other HS groups, which was also significantly lower than the control group (pH 5.95; p<0.01). The pH_24 h_ in the 38°C and 36°C groups both decreased to 5.87, but they had no differences with control group (p>0.05). HS time and the interaction with temperature had no effect on the pH values (p>0.05, Table 2).

Experiment pH results were agreement with most previous experiments. Broilers under HS condition at *antemortem* stage usually displayed a high pH drop rate at early *postmortem*, which finally contributed the development of PSE-like chicken meat. Garcia et al [27] found that the ultimate pH (5.67) in PSE-like chicken meat was significantly lower than normal meat (5.89). Wang et al [17] also found that the ultimate pH of HS (36°C, 1 h) group at 24 h *postmortem* was also much lower than the control group. Pan et al [13] and Tang et al [14] observed that broiler breast under 40°C had lower ultimate pH than controls, but they also found that as the HS time was extended to 10 h, the breasts’ ultimate pH value was higher.

The rapid pH decline in HS group may be caused by higher glycolysis enzyme activity at *postmortem*; the key glycolysis enzymes probably are activated by AMP-activated protein kinase (AMPK) in the early period after slaughter. A summer transportation experiment has revealed that broilers’ PM had higher p-AMPK level in the 0.5 h transportation group than in the control group [15]. For halothane positive Yorkshire pigs (higher frequency of PSE meat), the AMPK and Fructose 2,6-biphosphate (Fru-2,6-P2) activity from longissimus dorsi muscle was significantly higher than halothane negative Yorkshire pigs, which resulted in a higher pH drop rate from 0 h to 4 h *postmortem* [28].

### Meat quality

HS had no significant effect on L*_5 min_ or b*_5 min_, (p>0.05, Table 3) but significantly decreased a*_5 min_ (p<0.01, Table 3). Also, it increased the L*_24 h_ (p<0.01, Table 3) and decreased a*_24 h_ (p< 0.05, Table 3). L*_24 h_ in the 36°C group increased to 53.15, which was significantly higher than controls (50.56, p<0.01). However, counter to our expectation, when the HS temperature increased to 38°C or 40°C, the L*_24 h_ exhibited a statistically non-significant decline from 53.15 to 52.01 and 50.89 (Table 3). Also, at higher HS temperatures (38°C and 40°C), L*_24 h_ values (52.02 and 50.89, respectively) had no difference from the control (50.56; p>0.05). When HS temperature increased to 38°C or 40°C the significant increase in L*_24 h_ of 36°C disappeared. Pre-slaughter HS significantly decreased both a*_5 min_ (p<0.01) and a*_24 h_ (p<0.05). But there was no significant difference within HS groups (p>0.05). HS showed no significant effect on drip loss (p>0.05, Table 3) but HS temperature affected the cooking loss (p<0.05, Table 3), which significantly increased from 13.33% in the control group to 16.08% in the 36°C group (p<0.05). However, as the HS temperature increased to 38°C and 40°C, the cooking loss also decreased (14.15% and 13.79%) and the significant difference disappeared again (p>0.05). Time of HS within 2 hours and its interaction with HS temperature had no significant effect on meat quality (p>0.05). This indicated that the HS effect probably could be imposed in a very short time (less than 1 hour) as the prolongation of HS to 2 hours did not aggravate the negative effects on meat quality in this study.

Previous research indicated that HS (36°C, 1 h) significantly increased broiler breast L*_24 h_ [14]. For example, Sandercock et al [26] also pointed out that acute HS (32°C, 2 h) had adverse effects on breast meat quality [26]. The results of the 36°C group in the present study, agree with the above findings. However, when the HS temperature increased to the high levels of 38°C and 40°C, both the L*_24 h_ value and cooking loss began to decline, and the significant difference with the control group disappeared. These changes of meat quality under high temperature (>38°C) were in disagreement with some previous studies [9,29–31]. But some studies also reported a similar effect; Pan et al [13] and Tang et al [14] reported that broilers at 40°C HS had higher L* than controls, but as HS time extended to 10 h, the L* also became lower. Northcutt et al [12] also reported similar results. They found that a 40°C to 41°C HS treatment caused a lower cooking loss than controls. Both of the studies were also conducted under high temperature (above 40°C).

Based on these results, it is postulated that high HS level would probably cause an adverse effect on meat quality, but this still needs further research. Neither Pan nor Northcutt conducted further research on this factor. Only Pan et al [13, 32] pointed out that higher stress levels might lead broilers to have increased stress tolerance which might be an explanation. Our current experiment also supports this explanation, since stressed broilers in the present study had significantly higher blood cortisol level at the highest stress temperatures (38°C and 40°C; p<0.01, Table 1), as might be expected, since cortisol levels generally rise in animals subjected to various types of stress, enabling the animal to exhibit a temporary stress tolerance [33]. Blood cortisol is known to increase glycogen and protein lysis in the short term and to sustain blood glucose levels, which may play a role in lowering muscle pH under stress conditions, and maybe in reducing drip loss in PSE meat, and also perhaps in slowing the development of stress-related PSE syndrome. For example, Choi et al [34] found that muscle cortisol concentration was negatively associated with drip loss (r = −0.49, p<0.001), cooking loss (r = −0.27, p<0.05) and lightness (r = −0.24, p<0.05) in pork when investigating the effect of cortisol concentration on muscle fiber characteristics and the technological and sensory quality traits of the porcine *longissimus dorsi* muscle. Wang et al [35] also found that heat exposure could result in greater concentrations of serum cortisol which was involved mainly in carbohydrate, lipid and protein metabolism. So, the high cortisol concentration may be the reason for the phenomenon found in this study, but the specific mechanism is not clear at present and it still needs further investigation.

### Protein solubility

Protein solubility is used as a parameter of protein denaturation. In this experiment, HS temperature treatments had no significant effect on sarcoplasmic protein solubility in this study (p>0.05, Table 4), but it significantly affected the myofibrillar protein (p<0.05) and total protein solubility (p<0.01, Table 4). The myofibrillar protein solubility in 36°C and 38°C groups (103.41 and 109.49 mg/g, respectively) was much lower than in the control group (123.20 mg/g; p<0.05). But myofibrillar protein solubility of the 40°C group increased to 120.58 mg/g, which had no significant difference from the control. For total protein, the 36°C and 38°C treatment had the lowest solubility compared to other groups. Similar to myofibrillar protein solubility, the total protein solubility decreased in 36°C and 38°C treatments but increased to 208.42 mg/g in 40°C group. No significant difference was observed between the 40°C group and control group (p>0.05). The time of HS and its interaction with temperature had no effect on the protein solubility (p>0.05).

The protein denaturation level of breast meat is a proof for the variation of pH decline rate. Previous research showed that a higher rate of pH drop at early *postmortem* stage would result in more denaturation of sarcoplasmic and myofibrillar protein, and this would affect meat light reflection and WHC [31,36,37]. The myofibrillar and total protein concentration showed the same trends as color and cooking loss in this study. Table 2 shows that HS at 36°C had caused a greater rapid pH decline than other HS groups during the first hour *postmortem*, which probably caused this effect. And this probably was the reason why the 36°C HS group had the lowest protein solubility, which then led to the higher L*_24 h_ value and lower WHC.

### Ultra-structure

**Figure 1:**
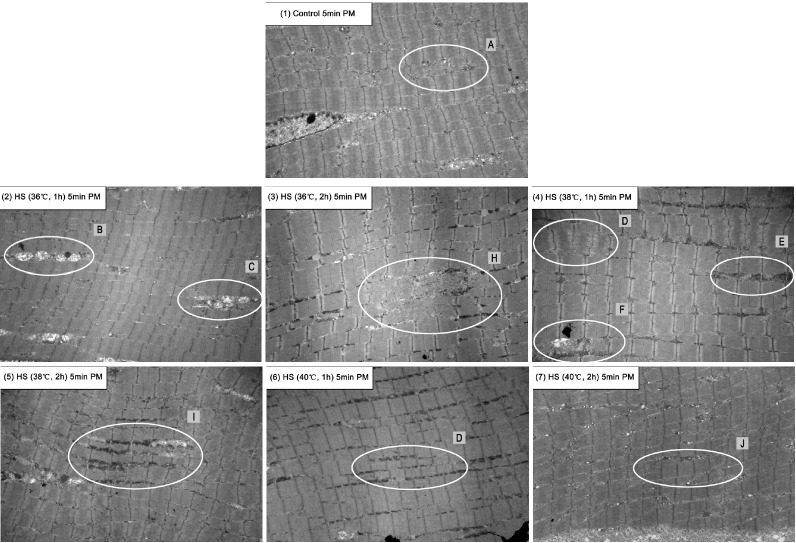
Effects of heat stress (HS) temperatures and exposure time on the ultra-structure of *pectoralis major* muscles at 5 min *postmortem* (10,000 magnification transmission electron micrographs). Picture (1), (2), (3), (4), (5), (6), and (7) showed the the ultra-structures of *pectoralis major* muscles at 5 min *postmortem* in control group, HS group (36°C, 1 h), HS group (36°C, 2 h), HS group (38°C, 1 h), HS group (38°C, 2 h), HS group (40°C, 1 h), and HS group (40°C, 2 h) respectively. The degradation of Z-lines and fuzz of H-zones in 36°C HS group (Picture (2) (3); C, H) was more serious than those of control group (Picture (1); A), and a significant degradation of intact sarcomeres in 36°C HS (Picture (3); 2 h, H) was observed, which was the most serious denature of all HS groups. Significant mitochondrial edemas were observed in 36°C HS (Picture (2); 1 h, B). As HS temperatures increased, the damage to mitochondria became subsided. Instead, significant degradations were observed between myofibrillar bundles (Picture (4)–(7); E, I, G, J) in 40°C HS groups.

**Figure 2:**
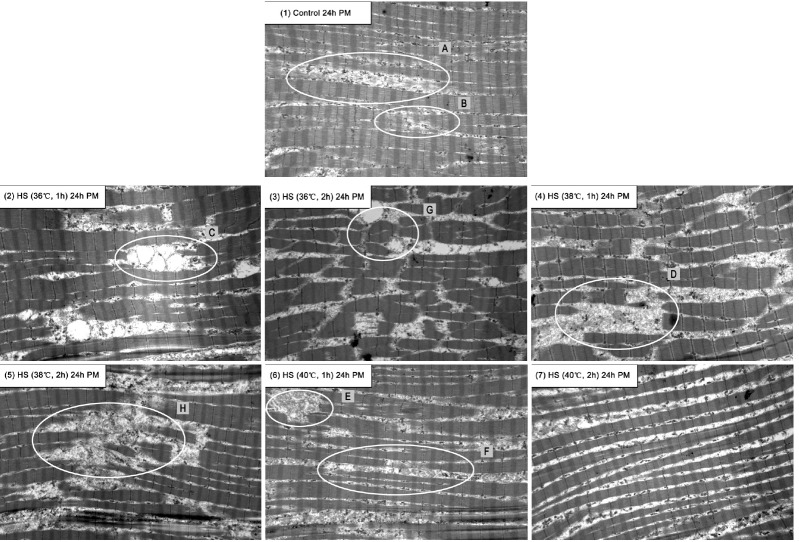
Effects of heat stress (HS) temperatures and exposure time on the ultra-structure of *pectoralis major* muscles at 24 h *postmortem* (10,000 magnification transmission electron micrographs). Picture (1), (2), (3), (4), (5), (6), and (7) showed the the ultra-structures of *pectoralis major* muscles at 24 h *postmortem* in control group, HS group (36°C, 1 h), HS group (36°C, 2 h), HS group (38°C, 1 h), HS group (38°C, 2 h), HS group (40°C, 1 h), and HS group (40°C, 2 h) respectively. The degradation of intact sarcomeres in HS (36°C, 38°C; Picture (2)–(5)) was more serious than other groups. Significant mitochondrial edemas in the degradation blanks were also observed in 36°C group (Picture (2); 1 h, C), and the most serious degration of myofibrils was found in 36°C HS group (Picture (3); 2 h). With HS exposure time increased to 2 h in 36°C group (Picture (3); G), the edema subsided and finally disappeared in 38°C HS group of (1 h, D). The detachments between myofibrillar bundles were more obvious (E, F) in 40°C group (40°C, 1 h and 2 h).

Transmission electron micrographs (Figures 1, 2) showed differences in ultra-structure with different HS temperatures and exposure time at 5 min and 24 h *postmortem*.

As can be seen in Figure 1, the degradation of Z-lines and fuzz of H-zones in 36°C HS (C, H) was more serious than in the control group (A) at 5 min *postmortem*. There was a significant degradation of intact sarcomeres in 36°C HS (2 h; H), which was the most serious denaturing in all HS groups. Significant mitochondrial edemas were observed in 36°C HS (1 h; B). As HS temperatures increased, the damage to mitochondria became less; instead, significant degradation was observed between myofibrillar bundles (E, I, G, J) in 40°C HS groups. In this case, HS did not result in much damage to muscle structure at *antemortem*. As HS temperatures increased and stress time was prolonged, HS contributed myofibrillar bundles detachments.

The difference between HS groups and control group was more apparent at 24 h *postmortem*. As shown in Figure 2, the degradation of intact sarcomeres in HS (36°C and 38°C) was more serious than other groups. Significant mitochondrial edemas in the degradation blanks were observed in 36°C group (1 h; C). Most myofibrillar structures in HS (36°C, 2 h) were seriously degraded. Samples exhibited fractured Z-lines, and overall myofibrillar structure was degraded (G). With HS time prolonged to 2 h (36°C, G), the edema subsided and finally disappeared at 38°C HS group (1 h, D). The detachments were more obvious in 40°C group (1 h and 2 h; E, F). These results indicated that HS at 36°C and 38°C had obvious myofibrillar degradation; it caused more damage of detachment of myofibrillar bundles at 40°C, with HS time also promoting these effects. The ultra-structure result showed that HS (36°C, 24 h) had significant mitochondrial swelling, which was an obvious damage reflection of stress condition [38]. However, as HS temperatures increased, this damage reflection disappeared.

These ultra-structure changes could probably explain the variation of meat color and WHC in different groups. As HS temperatures increased to 40°C, the myofibrillar fragmentation disappeared and each myofibril kept well. Instead, the detachment of myofibrillar bundles aggravated (Figure 1G, 1J; Figure 2E). The increasing gap between myofibrillar bundles might provide more valuable space for water and decrease the light reflection. This indicated that extremely high temperatures may change the muscle structure less than those of 36°C. This might present an explanation that HS at higher temperatures (40°C) did not increase the lightness and decrease the WHC.

## CONCLUSION

In conclusion, moderate high temperature (36°C) aggravated broilers’ meat quality via high glycolysis rate at early *postmortem* time, however, as the HS temperatures were raised to 40°C, the meat quality did not drop further, which probably can be explained by the changing of muscle structure. The mechanisms causing the effects of different HS levels on muscle structure changes are not clear for the moment. And further researches are needed to find the reason of much faster pH drop in 36°C group by investigating AMPK function and pathway changes in the *postmortem* glycolysis.

## Figures and Tables

**Table 1:** Effects of pre-slaughter heat stress temperature and exposure time on the initial core temperature of *pectoralis major* muscles and on the cortisol concentration in the blood (n = 12)

Item	Heat stress temperature (°C)					Heat stress time (h)			Significance		
		
	Control	36	38	40	SE	1	2	SE	Temp	Time	Temp×time
Temperature (°C)	41.57^b^	43.86^a^	44.14^a^	44.52^a^	0.19–0.22	43.40	43.64	0.14–0.15	**	NS	NS
Cortisol (nmol/L)	<0.50^c^	3.73^b^	5.93^a^	5.96^a^	0.45–0.53	3.66	4.25	0.32–0.35	**	NS	NS

SE, standard error; Temp, temperature; NS, no significance.

** p<0.01.

a–c Means in a row within different treatments with a different letter differ significantly.

**Table 2:** Effects of heat stress temperature and exposure time on the pH of *pectoralis major* muscles at 5 min to 24 h *postmortem*1) (n = 12)

*Postmortem* time	Heat stress temperature (°C)					Heat stress time (h)			Significance		
		
	Control	36	38	40	SE	1	2	SE	Temp	*P*time	Temp×*P*time
5 min	6.51	6.48	6.43	6.47	0.023–0.028	6.48	6.47	0.017–0.018	NS	**	**
1 h	6.36^a^	6.15^c^	6.27^b^	6.25^b^	0.020–0.024	6.25	6.27	0.015–0.016	**	-	-
2 h	6.17^x^	6.10^w^	6.13^x^	6.08^x^	0.022–0.027	6.12	6.12	0.016–0.018	NS	-	-
4 h	6.04^a^^y^	6.00^ab^^x^	6.01^a^^y^	5.94^b^^y^	0.020–0.019	5.99	6.00	0.014–0.015	**	-	-
24 h	5.95^a^^z^	5.87^ab^^y^	5.87^ab^^z^	5.79^b^^z^	0.022–0.026	5.88	5.88	0.016–0.017	**	-	-

SE, standard error; Temp, HS temperature; *P*time, *postmortem* time; NS, no significance.

1) The effect of HS time and the crossed effects of Temp×Time×*P*time and Time×*P*time were not significant (p>0.05).

** p<0.01.

a–c Means in a row within different treatments with a different letter differ significantly.

v–w Means a line within different postmortem time with a different letter differ significantly.

**Table 3:** Effects of pre-slaughter heat stress temperature and exposure time on the meat color and water holding capacity of pectoralis major muscles (n = 12)

Item	Heat stress temperature (°C)					Heat stress time (h)			Significance		
		
	Control	36	38	40	SE	1	2	SE	Temp	Time	Temp×time
L*_5 min_	49.01	48.86	48.67	48.15	0.43–0.45	48.48	48.86	0.30–0.32	NS	NS	NS
a*_5 min_	8.16^a^	7.05^b^	6.57^b^	7.06^b^	0.26–0.30	7.14	7.28	0.18–0.20	**	NS	NS
b*_5 min_	14.92	13.61	13.61	13.88	0.37–0.43	13.95	14.01	0.27–0.28	NS	NS	NS
L*_24 h_	50.56^b^	53.15^a^	52.02^ab^	50.89^ab^	0.35	52.26	51.77	0.25	**	NS	NS
a*_24 h_	9.85^a^	8.48^ab^	8.14^b^	9.38^ab^	0.40	8.88	9.04	0.28	*	NS	NS
b*_24 h_	16.39	15.82	15.45	15.75	0.39	15.90	15.81	0.28	NS	NS	NS
Drip loss (%)	1.10	1.40	1.20	1.17	0.12	1.22	1.21	0.08	NS	NS	NS
Cook loss (%)	13.33^b^	16.08^a^	14.15^ab^	13.79^ab^	0.61–0.66	13.94	14.72	0.44	*	NS	NS

SE, standard error; Temp, temperature; NS, no significance.

* p<0.05;

** p<0.01.

a–b Means in a row within different treatments with a different letter differ significantly.

**Table 4:** Effects of heat stress temperature and exposure time on the protein solubility of pectoralis major muscles (n = 12)

Items	Heat stress temperature (°C)					Heat stress time (h)			Significance		
		
	Control	36	38	40	SE	1	2	SE	Temp	Time	Temp×time
Sarcoplasmic protein (mg/g)	84.19	81.06	85.90	87.97	1.91–2.26	84.44	85.11	1.39–1.53	NS	NS	NS
Myofibrillar protein (mg/g)	123.20^a^	103.41^c^	109.49^bc^	120.58^ab^	3.29–3.89	112.43	115.92	2.42–2.64	*	NS	NS
Total protein (mg/g)	203.08^ab^	187.60^c^	190.11^bc^	208.42^a^	3.84–4.64	196.21	198.40	2.84–3.19	**	NS	NS

SE, standard error; Temp, temperature; NS, no significance.

* p<0.05;

** p<0.01.

a–c Means in a row within different treatments with a different letter differ significantly.
